# Surgical outcomes of robotic surgery for kidney stones: a systematic review and meta-analysis from section of YAU and EAU endourology

**DOI:** 10.1007/s00345-025-05734-x

**Published:** 2025-06-08

**Authors:** Rifat Burak Ergül, M. Fırat Özervarli, Frederic Panthier, Carlotta Nedbal, Arman Tsaturyan, Amelia Pietropaolo, Giovanni Cacciamani, Peter Kronenberg, Pieter De Backer, Chady Ghnatios, Zine-Eddine Khene, Sung Yong Cho, Ben Turney, Bhaskar Somani, Olivier Traxer, Tzevat Tefik

**Affiliations:** 1 Department of Urology, Istanbul Faculty of Medicine, Istanbul University, Istanbul, Turkey; 2 Department of Urology, Tenon Hospital, AP-HP, Paris, France; 3 GRC Urolithiasis 20, Sorbonne University, Paris, France; 4 PIMM Laboratory, UMR 8006 CNRS, Arts et Métiers Paris Tech, Paris, France; 5 Progressive Endourological Association for Research and Leading Solutions, Paris, France; 6 Department of Urology, ASST Fatebenefratelli Sacco, Milan, Italy; 7 Polytechnic University of Le Marche, Ancona, Italy; 8 Department of Urology, Erebuni Medical Center, Yerevan, Armenia; 9 Department of Urology, University Hospital Southampton NHS Foundation Trust, Southampton, UK; 10 Department of Urology, Keck School of Medicine, USC Institute of Urology and Catherine & Joseph Aresty, University of Southern California, Los Angeles, CA USA; 11 Department of Urology, Hospital CUF Descobertas, Lisbon, Portugal; 12 Department of Urology, ERN eUROGEN Accredited Centre, Ghent University Hospital, Ghent, Belgium; 13 Department of Mechanical Engineering, University of North Florida, Jacksonville, FL USA; 14 University of Texas Southwestern Medical Center, Dallas, TX USA; 15 Department of Urology, Seoul National University College of Medicine, Seoul National University Hospital, Seoul, 03080 Republic of Korea; 16 Department of Urology, Oxford University Hospitals NHS Foundation Trust, Oxford, UK

**Keywords:** Robotic endourology, Urolithiasis, Ureterorenoscopy, Retrograde intrarenal surgery

## Abstract

**Background:**

Urinary tract stone disease is quite common in the population, and treatment modalities are constantly evolving. Technological advancements in endourology have led to a shift towards more minimally invasive treatments. Nowadays, robotic flexible ureteroscopy is becoming increasingly popular, showing promising results. This systematic review and meta-analysis aimed to evaluate the outcomes of robotic flexible ureteroscopy.

**Materials and methods:**

We conducted a systematic review in the PubMed, Scopus and Web of Science databases based on the 2020 Preferred Reporting Items for Systematic Review and Meta-Analyses guideline. Study protocol was registered at PROSPERO (CRD420251017383). All robotic flexible studies published until April 2025, which defined and provided the stone-free rate, were included. To assess surgical efficacy and reliability, stone size, operation time, and complications were also evaluated. Stone size was measured in a one-dimensional manner, based on the maximum length.

**Results:**

A total of 320 studies were initially identified, with 11 full-text articles meeting the inclusion criteria, involving 656 patients and 660 renal units. The analysis included data from various robotic systems, including the Roboflex Avicenna, ILY, Senhance, and MONARCH platforms. The mean pooled stone-free rate was 86.0%, with a range from 57.7 to 96.5%, indicating variability across studies. The use of a random-effects model was justified by the presence of moderate-to-substantial heterogeneity across studies (I² = 63.5%, τ² = 0.627), and a statistically significant Q-test (*p* = 0.0022). The studies defined stone-free status as either complete stone clearance or residual fragments smaller than 2 mm.

**Conclusion:**

The analysis suggests that robotic URS is an effective and feasible treatment option for selected patients with urinary stones. Future research should focus on standardized reporting, comparative effectiveness studies, and cost-benefit analyses, while also addressing surgeon-centered outcomes such as ergonomic strain and musculoskeletal pain, to better define the role of robotic technology in endourological practice.

**Supplementary Information:**

The online version contains supplementary material available at 10.1007/s00345-025-05734-x.

## Introduction

Urinary stone disease affects 10% of the global population, making it the most common disorder of the urinary system [1]. Various surgical treatment modalities have been developed for its management, including open surgery, laparoscopic procedures, and endoscopic techniques [2]. However, with recent advancements in endourology, open and laparoscopic approaches have become less commonly used in the treatment of urolithiasis [3].

As minimally invasive surgery has continued to evolve, endourological techniques have become the cornerstone of contemporary stone management. Flexible ureteroscopy (F-URS), in particular, has gained wide acceptance due to its ability to access all regions of the collecting system with minimal morbidity [4]. Nonetheless, F-URS presents notable ergonomic challenges for the surgeon, especially during prolonged procedures or in patients with high stone burden [5]. To address these challenges, recent years have seen growing interest in the use of robotic assistance during F-URS. The goal is to enhance surgical precision, reduce operator fatigue, and ultimately improve patient outcomes.

Early attempts to integrate robotic systems into ureteroscopy began with the introduction of robotic assisted ureteroscopy (robo-URS) by Desai et al. in 2008 through an 18-case series using the Sensei^®^ robotic catheter system. This approach was designed to improve success rates, minimize complications, enhance surgeon comfort, and reduce radiation exposure [6, 7]. Since then, advances in robotic technology have led to systems with greater dexterity and control, allowing surgeons to address stones of various sizes and locations with the potential for complete clearance in a single session.

Although numerous preclinical and clinical studies have investigated this technique, a comprehensive synthesis of surgical outcomes associated with robo-URS is lacking. Therefore, this systematic review and meta-analysis aim to evaluate the current literature on surgical outcomes following robot-assisted flexible ureteroscopy and to provide pooled estimates of its safety and efficacy.

## Materials and methods

### Design and registration

This systematic review and meta-analysis were conducted in accordance with The Cochrane Handbook for Systematic Reviews of Interventions and the Preferred Reporting Items for Systematic Reviews and Meta-analyses (PRISMA) guidelines [8]. The completed PRISMA 2020 checklist is provided as Supplementary Table 1. The protocol was registered in the International Prospective Register of Systematic Reviews (PROSPERO) database with ID “CRD420251017383”.

### Information sources and search strategy

A comprehensive search was conducted across the following databases: PubMed, Scopus and Web of Science. Different combinations of the following keywords were used to search for articles by title/abstract: “Percutaneous Nephrolithotomy”, “Ureteroscopy”, “Flexible Ureteroscopy”, “F-URS”, “URS”, “PCNL”, “ECIRS”, “Intrarenal Surgery”, Robotic”, “Stone”, “Stones”, “Urolithiasis”, “Nephrolithiasis”, “Lithiasis”. The search was limited to studies published up to April 2025. The search strings are reported in Supplementary Table 2.

Once the identified studies were imported into Rayyan software [9], duplicate publications were detected and subsequently excluded from the analysis.

### Study selection

Two independent reviewers (R.B.E. and M.F.O.) screened the titles and abstracts of all identified studies. Full-text articles were reviewed for studies that met the eligibility criteria. During this process, conflicts or discrepancies were settled by agreement with the supervisor (T.T.).

The systematic review and meta-analysis were designed using the PICOS framework to define the scope and inclusion criteria for evaluating the robotic surgical outcomes of urinary system stone disease:

#### Population (P)

Patients of all ages with stones in the ureter or kidney.

#### Intervention (I)

Robot-assisted flexible URS/RIRS procedure.

#### Comparator (C)

Not applicable (both comparative and non-comparative studies included).

#### Outcome (O)

Stone-free rate, total operative time, robot docking time, complication rate.

#### Study design (S)

Prospective and retrospective comparative or noncomparative studies.

### Inclusion and exclusion criteria

The inclusion criterion for this study was defined as patients who underwent ureteral or kidney stone surgery using robot-assisted ureteroscopy (Robo-URS), with the stone-free rate demonstrated by any postoperative imaging technique. Studies that involved robot-assisted ureteroscopy with components such as a master console where the surgeon controls the procedure, a robotic manipulator or arm that holds and guides the flexible ureteroscope, and an integrated imaging system that provides real-time endoscopic visualization were included. The stone-free rate was defined as the success of the surgical method and used for comparison. Only Robo-URS techniques were included, while robotic PNL or robotic laparoscopic methods were excluded from the study. Therefore, studies with a clearly defined description of the stone-free status were included to analyze the success of the procedure.

Reviews, meta-analyses, editorial comments, letters or conference abstracts, and case reports were excluded. Studies in languages other than English and preclinical studies were also excluded from the meta-analysis.

### Data extraction

Data extraction was performed using Excel, where a structured table was created to compile key study characteristics, including author, year of publication, number of participants, patient sex, patient age, stone size, robot docking time, total operation time, definition of stone free status, success rate, and reported complications. Stone size was measured in a one-dimensional manner, based on the maximum length.

Each reviewer (R.B.E and M.F.O.) independently extracted the data, and the results were subsequently cross-checked to ensure accuracy and eliminate potential errors. No assumptions or simplifications were made during the extraction process. All relevant data were obtained directly from the included studies, and it was not necessary to contact any authors for clarifications or additional information.

### Quality assessment

The risk of bias was independently assessed by two authors (R.B.E and M.F.O.) using the ROBINS-I tool for non-randomized trials (Supplementary Table 3) and the Cochrane Risk of Bias 2 tool for randomized controlled trials (RCTs) (Supplementary Table 4). In cases of disagreement, a third author (T.T.) was consulted to help reach a consensus.

### Statistical analysis

This systematic review and meta-analysis synthesized data from published studies evaluating stone-free rates following robo-URS procedures. All statistical analyses were performed using R software (version 4.4.2).

For studies in which only the median values of variables such as age, stone size, robot docking time, or total operative time were reported, the corresponding mean values were estimated using the method proposed by Hozo et al. (2005) [10]. These estimated means were subsequently incorporated into the weighted average calculations to derive the overall mean values for each parameter.

In the first phase of the analysis, the metafor package was used to estimate pooled logit-transformed success rates through a random-effects model, employing the restricted maximum likelihood (REML) method to estimate between-study variance. Forest plots were generated using the forest() function, with study labels (slab) and the x-axis labeled as “Logit-Transformed Success Rate.”

In the second phase, the meta package was utilized to perform a meta-analysis of proportions using the metaprop() function. The effect size was defined as a logit transformation (“PLOGIT”), and a generalized linear mixed model (GLMM) was applied. Between-study heterogeneity was estimated using the maximum likelihood (ML) method. A random-effects model was adopted when heterogeneity was present, while a fixed-effect model was used in the absence of heterogeneity. Prediction intervals were also computed and visualized using forest plots with customized color schemes.

A random-intercept logistic regression model was used to model between-study variance (τ²) under the random-effects framework. Individual study estimates were calculated using the Clopper-Pearson method, and a continuity correction of 0.5 was applied in studies with zero cell frequencies, where necessary.

## Results

**Fig. 1:**
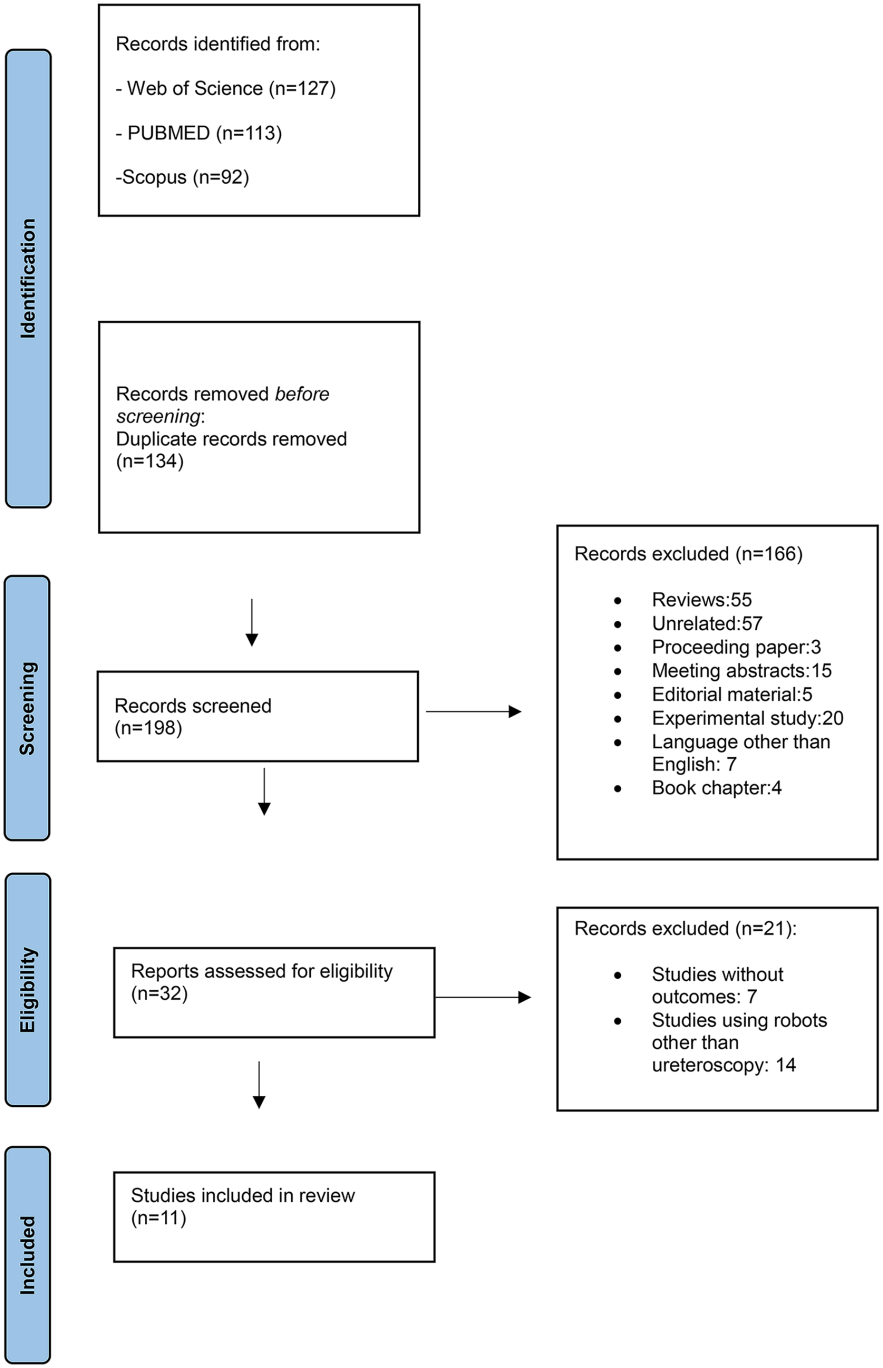
Prisma flowchart adapted for the selection of studies included

A total of 320 studies were initially identified from a literature search across 3 databases. After removing 134 duplicate studies, 198 studies were screened, and 55 reviews, 57 unrelated studies, 3 proceeding papers, 15 meeting abstracts, 5 editorial materials, 20 experimental studies, 7 studies in languages other than English, and 4 book chapters were excluded. When assessed according to eligibility, 7 studies were excluded due to the absence of outcomes, and 14 studies were excluded because they used robots other than ureteroscopy. Finally, 11 full-text articles met the inclusion criteria and were included in the meta-analysis (Fig. 1).

**Fig. 2:**
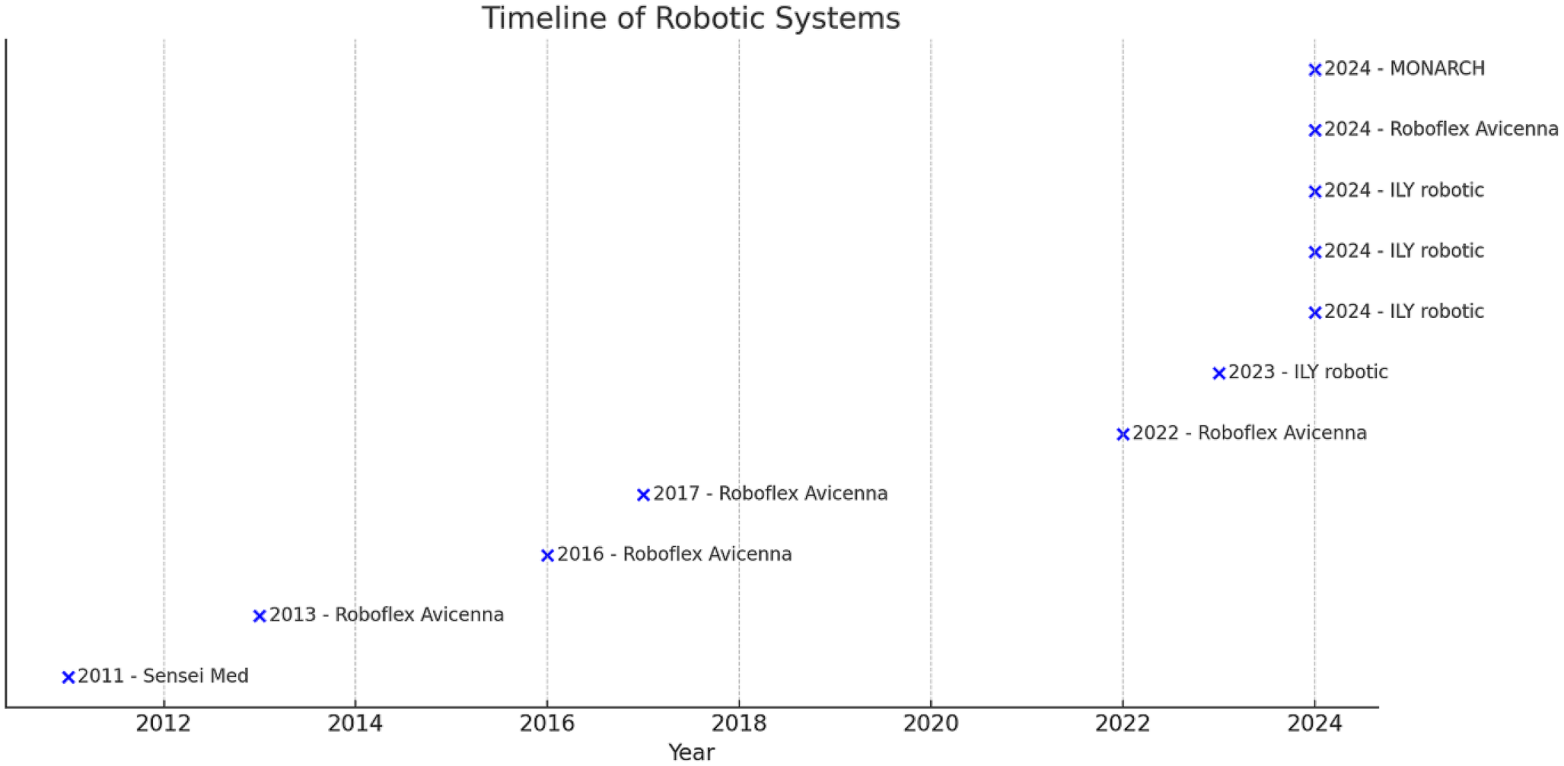
Timeline of robots as introduced and published in literature

Among the studies included in the review, five involved the Roboflex Avicenna system, four used the ILY robotic system, while one study each reported outcomes using the Senhance system and the MONARCH platform (Fig. 2). A total of 529 procedures were performed using the Roboflex Avicenna system, 96 with the ILY robotic system, 18 with the Senhance system, and 13 with the MONARCH platform (Supplementary Fig. 1).

A total of 656 patients were included, with 237 females and 315 males, while the gender of 104 patients was not specified and therefore could not be presented. The overall mean age, stone size, robot docking time, and total operation time were estimated as 47.9 years, 15.8 mm, 272.8 s, and 90.5 min, respectively. Among the 208 patients who underwent robotic surgery and were evaluated in the preoperative period, 116 (55.8%) had a JJ stent in place prior to surgery. Postoperative JJ stent data were available for 650 patients; of these, only 19 (2.9%) did not have a stent placed following the procedure. Additionally, among 590 patients, a ureteral access sheath was not used in only 28 cases (4.7%) (Table 1).

**Table 1:** Table summarizing all the studies included

	Author-Year	Operation Method	Patients/*n* (female/male)	Age/years	Robotic System	Stone size	Pre-Stented (*n*)	Post-Stented (*n*)	Acces Sheath (patients (size, Fr))	Robot Docking Time	Total Operation Time	Definition of Stone Free Status	Stone Free Rate, %	Complications
**1.**	Desai et al.- 2011 [6]	Robo-URS	18 (6/12)	46 (26–74) *	Sensei Med	11.8 mm (9–25) *	18/18	18/18	18/18 (12/14)	7.3 min (4–18) *	91.3 min (60–130) *	2 months postoperative on CT No RF	56% (10/18)	-No intraoperative complications -Transient upper extremity paresis in patient with severe kyphoscoliosis: 5.5% (1/18) -Febrile urinary tract infection that responded to antibiotics. 11,1% (2/18)
												3 months postoperative on IVP No RF	89% (15/18)	
**2.**	Saglam et al. − 2013 [29]	Robo-URS	81 (25/56)	42 (6–68) * SD: 25.4	Roboflex Avicenna	13 mm (5–30) * SD: 5.3	N/A	81/81	72/81 (10/12)	59.6 Sects. (35–124) * SD:45	74 min (40–182) * SD:31.8	3 months postoperative on X-ray and USG No RF	80% (65/81)	-Failure of endoscope classic FURS not possible; placement of DJ stent:1,2% (1/81)
**3.**	Gaevlete et al. − 2016 [21]	Robo-URS	66 (39/27)	51 (25–74) *	Roboflex Avicenna	24 mm (10–37) *	N/A	66/66	N/A (N/A)	-	51 min (38–103) *	3 months postoperative No RF	92.4% (61/66)	-No intraoperative complications
		Conventional-F URS	66 (39/27)	48 (26–77) *		21 mm (11–36) *		66/66	N/A (N/A)		50 min (41–115) *	3 months postoperative No RF	89.4% (59/66)	-Intraoperative bleeding: 1.5% (1/66)
4.	Klein et al.– 2017 [25]	Robo-URS	240 (100/140)	55.7 (18–87) * SD: 17.24	Roboflex Avicenna	14 mm (5–30) *	N/A	240/240	240/240 (12/14)	4 min (1–29) *	96 min (59–193) *	3 months postoperative on X-ray and/or USG ≤2 mm RF	90% (216/240)	- Perioperative complications: 5.4% (13/240) -Bad vision: 0.4% (1/240) -Technical errors of the system requiring conversion to conventional F-URS: 0.4% (1/240) -Mucosal bleeding: 2.5% (6/240) -Ureteral lesions caused by the placement of the UAS: 0.8% (2/240) -Perforation of calyx and stone dislocation:0.8% (2/240) -Subcapsular renal hematoma: 0.4% (1/240)
5.	Tokatli et al.– 2022 [14]	mini-ECIRS	42 (18/24) (44 renal units)	42.3 ± 12.8 **	Roboflex Avicenna	28.4 +/–4.6 mm **	N/A	42/42	42/42 (10.7/12.7)	-	103.7 ± 20.6 min **	1 month postoperative on CT No RF	95.5% (42/44)	-Fever 4.8% (2/42) -Hematuria 2.3% (1/42)
6.	Fiori et al.– 2023 [12]	Robo-URS	4 (N/A)	57 (49–61) *	ILY robotic	13 mm (10–15) *	1/4	4/4	4/4 (10/12)	3 min (3–4)*	70 min (61–94) *	1 month postoperative on USG No RF	100% (4/4)	-No perioperative complications
7.	Salah et al.– 2024 [30]	Robo-URS	100 (N/A)	40.7 ± 9.2 **	Roboflex Avicenna	11.7 ± 5.8 mm **	58/100	100/100	100/100 (N/A)	7.8 ± 3.2 min **	116 min (97–148) *	3 months postoperative on CT; Grade A no RF	Grade A 73% (73/100)	-A total of 8 complications were recorded in 5 patients 5% (5/100) -Ureteral injury with extravasation of contrast: 1% (1/100) -Urinary tract infection: 2% (2/100) -Fever 2% (2/100) -Stent related discomfort 2% (2/100) -Hematuria 1% (1/100)
												Grade B RF ≤ 2 mm	Grade B 78% (78/100)	
8.	Laszkiewicz et al. 2024 [11]	Robo-URS / mini-ECIRS	57 (29/28) (Robo-URS/mini ECIRS: 46/11)	46 (18–82) * SD: 19.3	ILY robotic	Robo-URS 13 mm. (8–23) *	32/46	31/46	34/46 (10.7/12.7)	Docking (with UAS) 73 Sects. (32–124) * Docking (without UAS) 61 Sects. (30–99) *	Robo-URS 63 min (15–91) *	Perioperative endoscopic and fluoroscopic evaluation revealed no fragments > 2× laser fiber diameter.	Robo-URS 80.4% (37/46)	N/A
						mini-ECIRS 19 mm (11–56) *	5/11	7/11	4/11 (10.7/12.7)		mini ECIRS 55 min (32–83) *		mini-ECIRS 90.9% (10/11)	
9.	El-Hajj et al.– 2024 [15]	Robo-URS	29 (10/19) (31renal units)	56 (44.5–64.5) ***	ILY robotic	13 mm (12–20) ***	2/29	29/29	29/29 (N/A)	3.5 min (3–5) ***	85 min (60.5–100) ***	2 or 3 weeks postoperative on X- ray or CT No RF	93.55% (29/31)	-No perioperative complications -Pain-related visits to the emergency department that did not require hospital admission: 9.68% (3/29)
10.	Farre et al.– 2024 [31]	Robo-URS	6 (3/3)	62 (50–72) *	ILY robotic	13.5 mm (11–15) ***	N/A	N/A	6/6 (10/12)	-	77.5 min (65–90) ***	3 months postoperative on CT ≤2 mm RF	83.3% (5/6)	-Fever treated with antibiotics: 16,7% (1/6) -Underwent an uncomplicated infundibulotomy for intradiverticular stone developed a pseudoaneurysm non-attributed to the use of the robot, which was managed with selective embolization: 16,7% (1/6)
11.	Landman et al.– 2024 [13]	Robotic Assisted Platform for Combined Mini-Percutaneous Nephrolithotomy and Flexible Ureteroscopic Lithotripsy	13 (6/7)	65 (35–72) ***	MONARCH	32.8 mm (11.8–65.2) ***	N/A	13/13	12/13 (12/14) 1/1 (10/12)	Robotic retrograde access docking time: 9 min (4–19) *** Robotic antegrade access docking time: 10 min (8–30) ***	183 min (83–383) ***	1 month postoperative on CT; Grade A no RF	Grade A 38.5% (5/13)	- Postureteroscopic lesion scale grade 2 ureteral wall injury: 7.7% (1/13) - Urinary tract infection: 7.7% (1/13) - Symptomatic urinary tract infection requiring hospitalization: 7.7% (1/13)
												Grade B RF ≤ 2 mm	Grade B 46,1% (6/13)	
												Grade C RF ≤ 4.0 mm	Grade C 61,5% (8/13)	

*: mean (range)

**: mean ± standard deviation

*** median (range)

Robo-URS: Robotic Ureterorenoscopy

mini-ECIRS: mini- Endoscopic Combined Intrarenal Surgery

SD: standard deviation

min: minute

sec: second

CT: Computed Tomography

IVP: Intravenous Pyelography

USG: Ultrasonography

RF: Residual Fragments

UAS: Ureteral Access Sheath

A total of 11 studies, comprising 660 renal units (from 656 patients) and 568 stone-free events, were included in this meta-analysis (Table 1). Stone-free rates were assessed across all included studies. The study by Laszkiewicz et al. [11] assessed outcomes in the perioperative period, while the studies by Fiori [12], Landman [13], and Tokatli et al. [14] evaluated patients at postoperative month 1. El-Hajj et al. [15] assessed stone-free status at 2–3 weeks postoperatively. The remaining studies evaluated stone-free rates at postoperative month 3. In all included studies, stone-free status was defined as complete stone clearance or residual fragments smaller than 2 mm. In cases where both criteria were reported, residual fragments smaller than 2 mm were selected for inclusion in the meta-analysis.

**Fig. 3:**
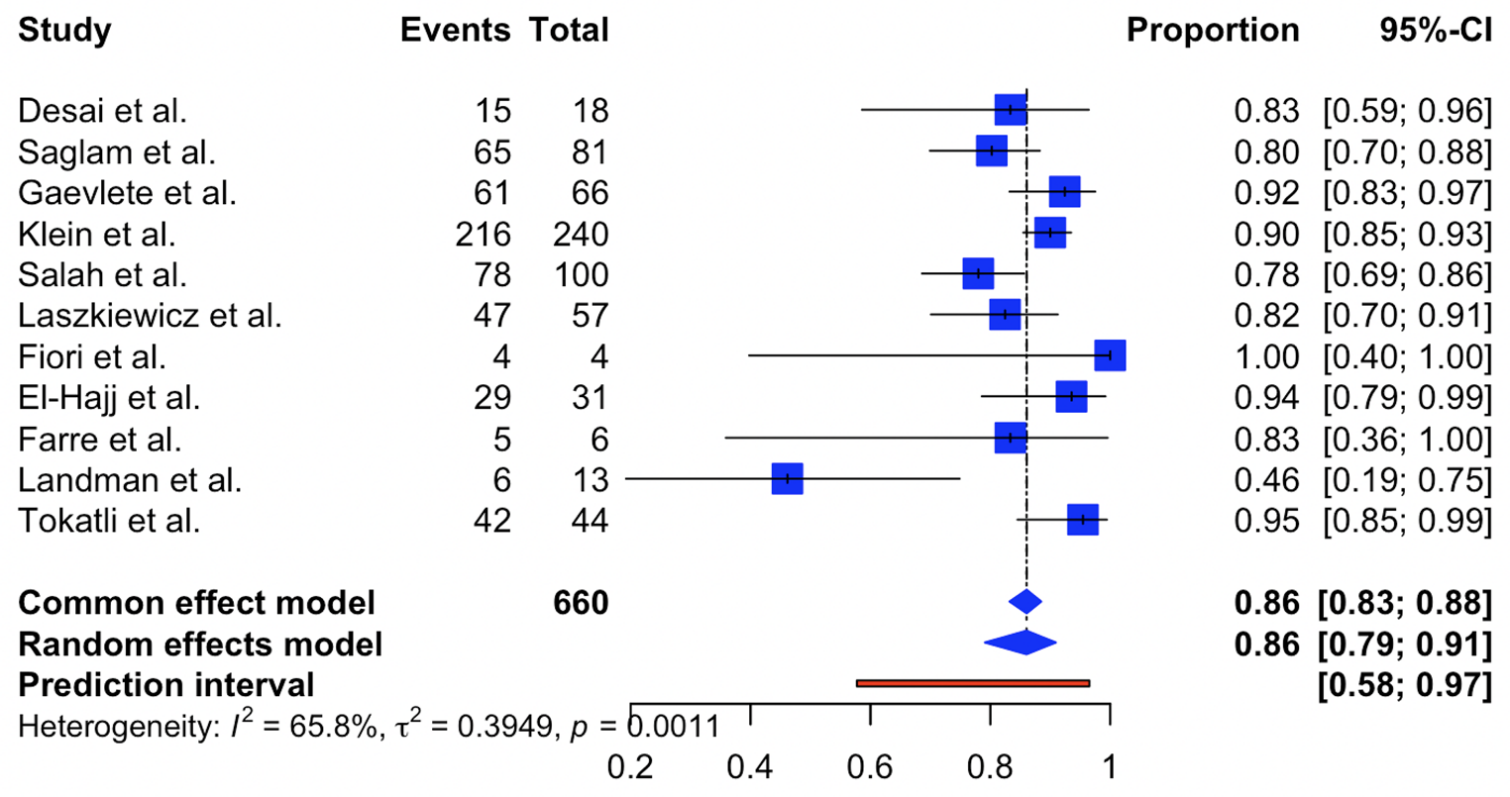
Meta-analysis of stone free rate of robot assisted ureterorenoscopy

Due to moderate-to-substantial heterogeneity among studies (I² = 65.8%, 95% CI: 35.2–82.0%; τ² = 0.3949) and a statistically significant heterogeneity test (Q = 29.24, df = 10, *p* = 0.0011), a random-effects model was applied, which accounts for potential variability in true effect sizes across studies. The pooled stone-free rate was estimated to be 86.0% (95% CI: 79.1–90.9%). The prediction interval ranged from 57.7 to 96.5% (Fig. 3). Among the included studies, the highest stone-free rate was reported by Tokatli et al. (95.5%) [14], whereas the lowest was observed in Landman et al. (46.2%) [13].

## Discussion

This meta-analysis demonstrates that robo-URS is associated with a high overall stone-free rate, with a pooled estimate of 87.1% based on a random-effects model. This finding highlights the promising efficacy of robo-URS in the management of urolithiasis. The use of a random-effects model was justified by the presence of moderate-to-substantial heterogeneity across studies (I² = 63.5%, τ² = 0.627), and a statistically significant Q-test (*p* = 0.0022). The prediction interval ranged from 50.6 to 97.8%, indicating potential variability in clinical outcomes across different settings and patient populations.

The high success rates observed in most included studies are in line with recent advancements in robotic endourology, which offer enhanced precision, improved ergonomics, and potentially lower complication rates compared to conventional techniques [16]. However, the wide prediction interval and variability between studies—ranging from 46.2–95.5%—suggest that procedural success may still be influenced by factors such as stone burden, surgeon experience, case selection, and institutional resources.

The robotic flexible URS, initially applied by Desai et al., was actually the Sensei-Magellan system designed for angiography and transvascular cardiologic interventions. Due to its use outside its intended purpose, the device caused technical issues during manipulation, leading to the discontinuation of its use [6, 17]. Later, to eliminate these disadvantages, the Avicenna Roboflex system was developed specifically for F-URS. With a rotation range of 210° in each direction (420° total), it provides a significant ergonomic advantage compared to classical FURS [17]. In the video article presented by Ergul et al., the effectiveness and feasibility of the Avicenna Roboflex were demonstrated [18]. In this meta-analysis, the Avicenna system was used in 529 patients, the ILY system in 44 patients, the Sensei Med system in 11 patients, and the Monarch platform in 11 patients. Although these platforms differ in their technical design and functionality, a direct comparison was not feasible due to heterogeneity among studies and variability in outcome reporting. (Supplementary Fig. 1).

Although the stone-free rate of robotic flexible URS is high, similar rates have been observed in studies conducted with conventional flexible URS in the literature. In the meta-analysis by Aboumarzouk et al., involving 445 patients with stones of 2 cm<, the mean stone-free rate was found to be 93.7% (77–96.7%) [19]. In the study conducted by Frankhauser et al., the success rate of F-URS for stones less than 2 cm was found to be 84% [20]. In this meta-analysis, only the randomized controlled trial by Geavlete et al. was included, where two groups of 61 patients each were compared. After 3 months, the stone-free rate was 92.4% in the robotic group compared to 89.4% in the conventional group [21]. Although the robotic group’s rate was slightly higher, it is noteworthy that the rates were quite similar.

With an average stone size of 15.8 mm and an average total operation time of 90.5 min, robotic flexible URS shows a longer operation time compared to the conventional method. In a similar study with comparable stone sizes, the total operation time for conventional RIRS in 28 patients was reported to be 64.5 min [22]. Due to the learning curve associated with robotic surgery, it is expected that the operation time will be longer in early series. Additionally, the docking time and preparation process further increases the total duration compared to the conventional method [14].

In our study, the longest operation time of 183 min (83–383) is attributed to the work of Landman et al. The reason for this could be that the MONARCH Platform is the first to combine both PCNL and URS into one system for performing endoscopic combined intrarenal surgery (ECIRS), and the stone size, which was 32.8 mm (11.8–65.2), is higher than in other studies [13].

In a large series of 11,885 patients who underwent ureteroscopy, conducted by the Clinical Research Office of the Endourological Society, the most common intraoperative complications were bleeding (1.4%) and perforation (1.0%), while ureteral avulsion occurred in 0.1% of patients. The postoperative complication rate was low at 3.5%, with fever reported in 1.8% of patients, which is consistent with findings from other studies, the rates of Clavien III and IV were just 0.5% and 0.1%, respectively [23, 24]. In this meta-analysis, although the complication rate was higher in some studies, the small sample sizes make it difficult to perform a statistically significant evaluation. In contrast, the low incidence of grade 3 and higher complications according to the Clavien-Dindo classification is consistent with the literature. In the study by Landman et al., although complications occurred in 3 out of 13 patients (23.1%)—with one case of ureteral wall injury and two cases of infection—it is notable that no grade 3 or higher complications were reported [13]. In the study by Klein et al., which had the largest number of patients, complications occurred in 13 out of 240 patients (5.6%), with only 3 of them classified as grade 3 or higher according to the Clavien-Dindo classification [25].

To our knowledge, this is the first meta-analysis focused specifically on robo-URS. Our findings contribute to this growing body of literature and underscore the need for prospective, high-quality trials comparing robotic URS with other endourological approaches.

This study has several limitations. First, the total number of included studies (*n* = 11) was relatively small, and most were single-center retrospective analyses, potentially introducing selection and reporting biases. Second, heterogeneity in definitions of “stone-free status” and follow-up protocols across studies may have affected the comparability of outcomes. Third, the included studies varied in terms of robotic platforms and surgical expertise, which may have contributed to the observed heterogeneity. Additionally, two of the included studies did not report the gender of the patients, reflecting a lower reporting standard. While we chose to include these studies in order to maximize the available data and better assess surgical success, this limitation should be taken into consideration when interpreting the findings. Furthermore, the methodological rigor of the included robotic URS studies was generally lower than that of classical URS literature, with limited prospective or comparative designs. This variation limits the strength of cross-technique comparisons and highlights the need for more standardized, high-quality trials in robotic URS.

Ultrasound has a reported sensitivity of approximately 45% and specificity of 88% for detecting renal stones, while KUB has a sensitivity ranging from 44 to 77% [26, 27]. In contrast, a meta-analysis demonstrated that low-dose non-contrast CT detects urolithiasis with a pooled sensitivity of 93.1% and a specificity of 96.6% [28]. In our meta-analysis, 6 out of the 11 included studies used CT to assess stone-free status. Therefore, the use of different imaging modalities across studies may have impacted the accuracy of reported stone-free rates and represents a methodological limitation that should be considered when interpreting the results. Moreover, the higher sensitivity of CT, particularly in detecting small residual fragments, may lead to lower reported stone-free rates compared to studies that used ultrasound or KUB, which are less capable of identifying such small fragments.

Despite these limitations, our analysis suggests that robo-URS is an effective and feasible treatment option for selected patients with urinary stones. Future research should focus on standardized reporting, comparative effectiveness studies, and cost-benefit analyses to better define the role of robotic technology in endourological practice.

## Conclusion

This systematic review and meta-analysis demonstrate that robo-URS is a promising and effective approach for the treatment of renal and ureteral stones. With a high overall stone-free rate and a low incidence of high-grade complications, robo-URS appears to be a safe and feasible option, particularly in complex or high-burden stone cases. However, variability in surgical platforms, patient selection, and follow-up protocols across studies highlights the need for standardization in outcome reporting.

## Electronic supplementary material

Below is the link to the electronic supplementary material.

Supplementary Material 1

## Data Availability

No datasets were generated or analysed during the current study.
