# Supplementary material for: Surgical outcomes of robotic surgery for kidney stones: a systematic review and meta-analysis from section of YAU and EAU endourology
Source: World J Urol. 2025 Jun 8;43(1):364. doi: 10.1007/s00345-025-05734-x (PMC12146223; doi:10.1007/s00345-025-05734-x)
Supplement: Supplementary file 1 — Supplementary Material 1 [file 345_2025_5734_MOESM1_ESM.docx]

**Supplementary Table 1:** PRISMA 2020 Checklist

| **Section and Topic** | **Item #** | **Checklist item** | **Location where item is reported** |
| --- | --- | --- | --- |
| **TITLE** | | |  |
| Title | 1 | Identify the report as a systematic review. | Page 1 |
| **ABSTRACT** | | |  |
| Abstract | 2 | See the PRISMA 2020 for Abstracts checklist. | Page 1 |
| **INTRODUCTION** | | |  |
| Rationale | 3 | Describe the rationale for the review in the context of existing knowledge. | Page 3 |
| Objectives | 4 | Provide an explicit statement of the objective(s) or question(s) the review addresses. | Page 3 |
| **METHODS** | | |  |
| Eligibility criteria | 5 | Specify the inclusion and exclusion criteria for the review and how studies were grouped for the syntheses. | Page 4 |
| Information sources | 6 | Specify all databases, registers, websites, organisations, reference lists and other sources searched or consulted to identify studies. Specify the date when each source was last searched or consulted. | Page 5 |
| Search strategy | 7 | Present the full search strategies for all databases, registers and websites, including any filters and limits used. | Page 4 |
| Selection process | 8 | Specify the methods used to decide whether a study met the inclusion criteria of the review, including how many reviewers screened each record and each report retrieved, whether they worked independently, and if applicable, details of automation tools used in the process. | Page 4 |
| Data collection process | 9 | Specify the methods used to collect data from reports, including how many reviewers collected data from each report, whether they worked independently, any processes for obtaining or confirming data from study investigators, and if applicable, details of automation tools used in the process. | Page 5 |
| Data items | 10a | List and define all outcomes for which data were sought. Specify whether all results that were compatible with each outcome domain in each study were sought (e.g. for all measures, time points, analyses), and if not, the methods used to decide which results to collect. | Page 5 |
|  | 10b | List and define all other variables for which data were sought (e.g. participant and intervention characteristics, funding sources). Describe any assumptions made about any missing or unclear information. | Page 5 |
| Study risk of bias assessment | 11 | Specify the methods used to assess risk of bias in the included studies, including details of the tool(s) used, how many reviewers assessed each study and whether they worked independently, and if applicable, details of automation tools used in the process. | Page 6 |
| Effect measures | 12 | Specify for each outcome the effect measure(s) (e.g. risk ratio, mean difference) used in the synthesis or presentation of results. | Page 6 |
| Synthesis methods | 13a | Describe the processes used to decide which studies were eligible for each synthesis (e.g. tabulating the study intervention characteristics and comparing against the planned groups for each synthesis (item #5)). | Page 4 |
|  | 13b | Describe any methods required to prepare the data for presentation or synthesis, such as handling of missing summary statistics, or data conversions. | Page 6 |
|  | 13c | Describe any methods used to tabulate or visually display results of individual studies and syntheses. | Page 6 |
|  | 13d | Describe any methods used to synthesize results and provide a rationale for the choice(s). If meta-analysis was performed, describe the model(s), method(s) to identify the presence and extent of statistical heterogeneity, and software package(s) used. | Page 6 |
|  | 13e | Describe any methods used to explore possible causes of heterogeneity among study results (e.g. subgroup analysis, meta-regression). | Page 6 |
|  | 13f | Describe any sensitivity analyses conducted to assess robustness of the synthesized results. | Page 6 |
| Reporting bias assessment | 14 | Describe any methods used to assess risk of bias due to missing results in a synthesis (arising from reporting biases). | Page 6 |
| Certainty assessment | 15 | Describe any methods used to assess certainty (or confidence) in the body of evidence for an outcome. | Page 6 |
| **RESULTS** | | |  |
| Study selection | 16a | Describe the results of the search and selection process, from the number of records identified in the search to the number of studies included in the review, ideally using a flow diagram. | Page 12 |
|  | 16b | Cite studies that might appear to meet the inclusion criteria, but which were excluded, and explain why they were excluded. | Page 12 |
| Study characteristics | 17 | Cite each included study and present its characteristics. | Page 14-17 |
| Risk of bias in studies | 18 | Present assessments of risk of bias for each included study. | Supp figure 3 |
| Results of individual studies | 19 | For all outcomes, present, for each study: (a) summary statistics for each group (where appropriate) and (b) an effect estimate and its precision (e.g. confidence/credible interval), ideally using structured tables or plots. | Page 13 |
| Results of syntheses | 20a | For each synthesis, briefly summarise the characteristics and risk of bias among contributing studies. | Supp figure 3 |
|  | 20b | Present results of all statistical syntheses conducted. If meta-analysis was done, present for each the summary estimate and its precision (e.g. confidence/credible interval) and measures of statistical heterogeneity. If comparing groups, describe the direction of the effect. | Page 13 |
|  | 20c | Present results of all investigations of possible causes of heterogeneity among study results. | Page 8 |
|  | 20d | Present results of all sensitivity analyses conducted to assess the robustness of the synthesized results. | - |
| Reporting biases | 21 | Present assessments of risk of bias due to missing results (arising from reporting biases) for each synthesis assessed. | - |
| Certainty of evidence | 22 | Present assessments of certainty (or confidence) in the body of evidence for each outcome assessed. | Page 13 |
| **DISCUSSION** | | |  |
| Discussion | 23a | Provide a general interpretation of the results in the context of other evidence. | Page 8 |
|  | 23b | Discuss any limitations of the evidence included in the review. | Page 8-9 |
|  | 23c | Discuss any limitations of the review processes used. | Page 8-9 |
|  | 23d | Discuss implications of the results for practice, policy, and future research. | Page 8-10 |
| **OTHER INFORMATION** | | |  |
| Registration and protocol | 24a | Provide registration information for the review, including register name and registration number, or state that the review was not registered. | Page 4 |
|  | 24b | Indicate where the review protocol can be accessed, or state that a protocol was not prepared. | Page 4 |
|  | 24c | Describe and explain any amendments to information provided at registration or in the protocol. | - |
| Support | 25 | Describe sources of financial or non-financial support for the review, and the role of the funders or sponsors in the review. | Page 11 |
| Competing interests | 26 | Declare any competing interests of review authors. | Page 11 |
| Availability of data, code and other materials | 27 | Report which of the following are publicly available and where they can be found: template data collection forms; data extracted from included studies; data used for all analyses; analytic code; any other materials used in the review. | Page 11 |

*From:*  Page MJ, McKenzie JE, Bossuyt PM, Boutron I, Hoffmann TC, Mulrow CD, et al. The PRISMA 2020 statement: an updated guideline for reporting systematic reviews. BMJ 2021;372:n71. doi: 10.1136/bmj.n71. This work is licensed under CC BY 4.0. To view a copy of this license, visit <https://creativecommons.org/licenses/by/4.0/>

**Supplementary Table 2:** Search Strings

| Web of Science: | (TS=("Percutaneous Nephrolithotomy" OR "Ureteroscopy" OR "Flexible Ureteroscopy" OR "FURS" OR "URS" OR "PCNL" OR "ECIRS" OR "Intrarenal Surgery")) AND (TS=("robotic") AND (TS=("Stone" OR "Stones" OR "Urolithiasis" OR "Nephrolithiasis" OR "Lithiasis")) |
| --- | --- |
| PUBMED: | ("Percutaneous Nephrolithotomy"[All Fields] OR ("ureteroscopy"[MeSH Terms] OR "ureteroscopy"[All Fields] OR "ureteroscopies"[All Fields]) OR "FURS"[All Fields] OR "URS"[All Fields] OR "PCNL"[All Fields] OR "ECIRS"[All Fields] OR "intrarenal surgery"[All Fields]) AND ("robotic"[All Fields]) AND ("stone"[All Fields] OR "stones"[All Fields] OR "urolithiasis"[All Fields] OR "nephrolithiasis"[All Fields] OR "lithiasis"[All Fields]) |
| SCOPUS: | TITLE-ABS-KEY(Percutaneous Nephrolithotomy OR Ureteroscopy OR Flexible Ureteroscopy OR FURS OR URS OR PCNL OR ECIRS OR Intrarenal Surgery) AND TITLE-ABS-KEY(robotic) AND TITLE-ABS-KEY(Stone OR Stones OR Urolithiasis OR Nephrolithiasis OR Lithiasis) |

**Supplementary Table 3**. Risk of bias for non-randomized trials (ROBINS-I tool)

| Study ID | D1 | D2 | D3 | D4 | D5 | D6 | D7 | Overall |
| --- | --- | --- | --- | --- | --- | --- | --- | --- |
| Desai et al. |  |  |  |  |  |  |  |  |
| Saglam et al. |  |  |  |  |  |  |  |  |
| Klein et al. |  |  |  |  |  |  |  |  |
| Salah et al. |  |  |  |  |  |  |  |  |
| Laszkiewicz et al. |  |  |  |  |  |  |  |  |
| Fiori et al. |  |  |  |  |  |  |  |  |
| El-Hajj et al. |  |  |  |  |  |  |  |  |
| Farre et al. |  |  |  |  |  |  |  |  |
| Landman et al. |  |  |  |  |  |  |  |  |
| Tokatli et al. |  |  |  |  |  |  |  |  |

- Domain 1: Risk of bias due to confounding
- Domain 2: Risk of bias in classification of interventions
- Domain 3: Risk of bias in selection of participants into the study (or into the analysis)
- Domain 4: Risk of bias due to deviations from intended interventions
- Domain 5: Risk of bias due to missing data
- Domain 6: Risk of bias arising from measurement of the outcome
- Domain 7: Risk of bias in selection of the reported result

High Risk of Bias

Moderate Risk of Bias

Low Risk of Bias

**Supplementary Table 4**. Risk of bias for randomized controlled trials (Cochrane Risk of Bias 2 tool)

| Study ID | D1 | D2 | D3 | D4 | D5 | Overall |
| --- | --- | --- | --- | --- | --- | --- |
| Gaevlete et al. |  |  |  |  |  |  |

- Domain 1: Bias arising from the randomization process
- Domain 2: Bias due to deviations from intended interventions
- Domain 3: Bias due to missing outcome data
- Domain 4: Bias in measurement of the outcome
- Domain 5: Bias in selection of the reported result

High Risk of Bias

Moderate Risk of Bias

Low Risk of Bias

**Supplementary Figure 1:** The number of patients included in the meta-analysis according to the robot brand.
